# The Formative Development of the Sleep GOALS (Goal-focused Online Access to Lifestyle Support) Intervention for Postpartum Individuals: A qualitative study

**DOI:** 10.21203/rs.3.rs-8041369/v1

**Published:** 2025-11-23

**Authors:** Marquis S. Hawkins, Michele D. Levine, Daniel J. Buysse, Esa M. Davis, Rachel Wasilko, Mariska Goswami, Namhyun Kim, Eshika Kohli, Kathleen M. McTigue, Judy C. Chang

**Affiliations:** University of Pittsburgh; University of Pittsburgh; University of Pittsburgh; University of Maryland School of Medicine; University of Pittsburgh; University of Pittsburgh; University of Pittsburgh; Lake Erie College of Osteopathic Medicine at Seton Hill; University of Pittsburgh; University of Pittsburgh

**Keywords:** postpartum health, sleep intervention, multiple health behavior changes, qualitative research, lifestyle intervention development

## Abstract

**Background:**

While many perinatal weight loss interventions focus on caloric restriction and physical activity, few address sleep despite its independent effects on weight, diet, and physical activity and the high prevalence of sleep disturbances among postpartum individuals. This study aims to inform the program production stage of intervention development for an integrated sleep, diet, and physical activity intervention by identifying behavior change priorities, barriers, and delivery preferences.

**Methods:**

We conducted individual interviews with 20 postpartum individuals following a preinterview survey that was sent alongside a newsletter summarizing findings from an earlier phase of the study. The survey assessed the perceived importance and difficulty of intervention components related to sleep, diet, and physical activity. The interview guide was designed to (1) identify which behavioral domain participants viewed as most important to address first; (2) explore the perceived difficulty of changing each behavior; (3) determine which topics to adapt and include in the intervention; and (4) elicit reflections on the newsletter with earlier study findings. The survey responses were summarized descriptively, and the interviews were analyzed via qualitative content analysis to identify key themes.

**Results:**

Five themes emerged. (1) The participants affirmed that the newsletter reflected their experiences but identified gaps related to mental health and partner/provider support. (2) Sleep is viewed as the most important behavior to change, with strong support for strategies targeting sleep efficiency, sleep routines, and sleep-related worry. (3) Sleep is also perceived as the most difficult behavior to change due to infant-related sleep disruptions, parental fatigue, and limited knowledge of effective strategies. (4) Participants offered differing views on where to begin—some preferred starting with sleep, others preferred starting with diet and physical activity, or an integrated approach. (5) Participants emphasized the need for support and accountability, including coaching, peer connection, and flexible tools.

**Conclusion:**

The findings provide concrete guidance for tailoring intervention content, structure, and delivery to postpartum individuals’ values, constraints, and readiness for change.

## Backgrounds

Despite widespread efforts to promote postpartum weight loss through diet and physical activity interventions, success has been modest.(1–4) A growing body of evidence suggests that sleep health, although rarely integrated into these interventions, may play an important role in enhancing their effectiveness.(5, 6) Poor sleep health can undermine intervention effectiveness through its effects on appetite regulation, energy levels, and adherence to intervention protocols.(7–21) However, few postpartum interventions systematically address sleep.

There is evidence from clinical trials in non-perinatal populations that integrating sleep interventions into weight management programs can enhance outcomes. For example, the Better Weight-Better Sleep Study (BWBS) randomized participants to a diet and exercise weight loss intervention with or without an integrated sleep management component.(22) Compared with those in the diet and exercise intervention arm, the participants in the diet, exercise, and sleep intervention arm lost twice as much weight and reported greater improvements in coping self-efficacy. Other studies in general adult, child, and adolescent populations have reported similar results, showing that compared with interventions focused on diet and activity alone, integrated interventions targeting sleep, diet, and physical activity produce greater improvements in weight and health behaviors.(23–25) However, these studies did not include postpartum individuals—a population that is disproportionately burdened by poor sleep health—highlighting the need for tailored, integrated approaches that consider the unique challenges of the postpartum period.(26)

To begin developing a postpartum sleep, diet, and physical activity intervention, we conducted a formative qualitative study to understand postpartum individuals’ experiences with and perceptions of sleep, diet, and physical activity.(27) In this earlier study, semi-structured interviews with postpartum individuals explored the barriers to and facilitators of engaging in health-promoting behaviors, paying special attention to how sleep influences other lifestyle behaviors. The participants emphasized the need for integrated, flexible, and accessible interventions that reflect their lived experiences and support their evolving postpartum needs. Their feedback was the foundation for identifying two existing evidence-based programs to use as starting points for creating a tailored postpartum intervention.

The first evidence-based intervention identified was an online version of the Diabetes Prevention Program (DPP), which included behavioral (diet and physical activity) weight loss content, self-monitoring tools, personalized e-coaching, and links to reputable health resources.(28, 29) The DPP is an ideal starting point because it has been adapted and translated into a variety of community, clinical, and workplace settings.(30) The second was the Transdiagnostic Sleep and Circadian (TranS-C) intervention—a modular, evidence-based program that integrates cognitive and behavioral strategies to promote multidimensional sleep health.(31) TranS-C is particularly well suited for adaptation because it includes optional modules that can be flexibly selected on the basis of individual needs, and its modular structure allows for the addition of new content areas.

At this phase of our overall project, we now need to prioritize which of the DPP and TranS-C are perceived by postpartum individuals as most relevant to them. We thus conducted another qualitative interview study with postpartum individuals, asking them to help inform the program production stages of intervention development (i.e., refining materials, organization, and structure) by identifying behavior change priorities, barriers, and delivery preferences. The findings from this study will increase the interventions’ relevance and acceptance for postpartum individuals.

## Methods

### Study Overview

**Figure 1:**
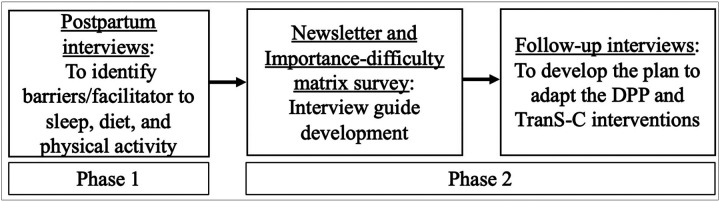
Overview of the multi-phase process used to develop the Sleep GOALS (Goal-focused Online Access to Lifestyle Support) intervention for postpartum individuals, including prior qualitative interviews, a participant newsletter with survey, and the current refinement interviews. DPP: Diabetes Prevention Program; TranS-C: Transdiagnostic Sleep and Circadian intervention.

Figure 1 provides an overview of this multi-phase process, beginning with prior qualitative interviews (27), followed by the newsletter and importance–difficulty survey, and culminating in the current set of interviews used to refine the intervention prototype. We used a qualitative descriptive approach grounded in naturalistic inquiry to explore how best to adapt existing evidence-based intervention content to meet the needs of postpartum individuals.(32, 33) As Sandelowski described, qualitative description seeks to provide a rich, straightforward account of participants’ experiences and perceptions via everyday language without imposing complex theoretical interpretations.(32) This approach is especially well suited for formative work aimed at intervention development because it allows for a clear and direct understanding of participants’ priorities, preferences, and perceived challenges.

In the context of our study, a qualitative descriptive framework enabled us to capture postpartum individuals’ perspectives on sleep, diet, and physical activity in their own words, providing practical insights that can inform concrete modifications to existing intervention materials. Rather than testing or developing new theories, our goal was to understand *what* matters most for postpartum individuals and *how* they believe that behavioral changes might be successfully integrated into their lives. This pragmatic lens is ideal for tailoring intervention content in ways that enhance acceptability, feasibility, and relevance for the end user.

### Study Population

Efforts were made to re-engage participants interviewed during the first stage of intervention development; however, challenges due to the pandemic also necessitated the recruitment of additional subjects. Some participants could not recall their prior interviews, and others were no longer interested. Therefore, we used a purposive, heterogeneous sampling approach to recruit individuals who met the same eligibility criteria as those in the first qualitative study—females who were at least 18 years of age, who gave birth in the previous year, and who were not currently pregnant at the time of the interview. Participants were recruited from Allegheny County, PA, via Pitt + Me, an extensive clinical research registry maintained by the University of Pittsburgh’s Clinical and Translational Science Institute,(34) and Pittsburgh Brown Mamas,(35) an online community group to support black mothers. Interested individuals were contacted by research team members who assessed eligibility and collected general demographic information (i.e., age, infant age, current pregnancy status). The participants gave verbal informed consent before data collection. Data were collected from August 2022 to January 2023. The University of Pittsburgh’s Institutional Review Board approved the study (Protocol # STUDY19110195).

### Interview Guide Development

We used a participatory and iterative process grounded in qualitative descriptions to develop the interview guide. Prior to the interviews, the participants received two materials: (1) a newsletter summarizing findings from our prior qualitative study (Appendix 1) and (2) a preinterview survey assessing the perceived importance of including intervention content across sleep, diet, and physical activity domains and the perceived difficulty of changing these behaviors (Appendix 2). The semi-structured interviews began with open-ended questions about the newsletter, followed by targeted discussion about survey responses to obtain additional context (see Appendix 3).

The newsletter served as a stimulus to promote reflection and discussion. Common themes identified in the earlier interviews, including the emotional and logistical challenges of postpartum sleep and the interrelationships between sleep, diet, and physical activity, were presented. The participants were asked to reflect on whether these themes aligned with their own experiences and to identify any perspectives that may have been missing.

The accompanying survey was a modified importance–difficulty matrix designed as a decision-support tool rather than a hypothesis test and was used to guide specific design decisions during adaptation of the DPP and TranS-C interventions. Its purpose was to identify which behavioral domains and specific sleep modules should be prioritized for adaptation. This matrix provided a structured way to capture participants’ perceptions of behavioral importance and difficulty in informing program sequencing, content emphasis, and coaching support. The participants rated (a) the importance of including intervention content and (b) the difficulty of changing behaviors via 5-point Likert scales (1 = not at all to 5 = extremely). In multi-behaviour interventions, sequencing can shape outcomes; interventions can begin by targeting the behavior viewed as most important to change, or that is the “gateway” for improving other behaviors. The survey allowed us to assess which behavioral domain—sleep, diet, or physical activity—participants perceived as the highest priority.

Second, the survey highlighted the perceived difficulty of changing each behavior, which informed the intervention in two possible ways: 1) prioritizing behaviors that are both important and perceived as easier to change (to build early momentum) or 2) providing coaching support focused on helping participants address behaviors that are important but difficult to change. Both approaches require clear insight into participants’ perceptions of behavioral importance and difficulty.

Finally, the survey was designed to support several decisions in adapting the Transdiagnostic Sleep and Circadian (TranS-C) intervention. The TranS-C offers several optional modules related to sleep hygiene, sleep efficiency, managing sleep-related worry, and understanding how sleep interacts with other behaviors. The survey results helped us prioritize which of these components postpartum participants thought would be most valuable.

### Data Collection

We conducted individual semi-structured interviews, each lasting between 30 and 60 minutes. All interviews were conducted remotely by the same trained interviewer (RW – female, MPH-trained qualitative methodologist). After each interview, the principal investigator (MSH, male, PhD-trained behavioral researcher) and the interviewer (RW) debriefed to reflect on emerging patterns and assess whether new insights were arising. We concluded recruitment when participants consistently described similar experiences and when no new concepts emerged, indicating thematic saturation.(36) All the interviews were audio-recorded and transcribed verbatim by TranscribeMe.

### Analysis

Two research assistants (RW and MG) independently coded the interview transcripts via NVivo to organize and manage the coding. RW and MG cocoded four transcripts and used Excel to manually document coding agreement. A Cohen’s kappa coefficient of 0.60 was calculated, indicating acceptable agreement. Following this initial reliability check, MG completed coding of the remaining transcripts.

Once data coding was completed, we conducted qualitative content analysis to describe the participants’ experience systematically.(37) We grouped similar codes into broader categories, organizing them around critical aspects of participants’ perspectives on behavior change priorities, barriers, and intervention delivery preferences. Theme development was iterative and team-based. The PI (MSH) and interviewer (RW) met regularly to review the coded data, discuss the category structure, and refine the emerging themes. Decisions about which categories were collapsed, elevated, or renamed were made with the goal of ensuring that final themes closely reflected participants’ language and could meaningfully inform intervention development.

## Results

We conducted twenty semi-structured interviews with postpartum individuals. The participants had a median age of 33.0 years (IQR: 29, 35), and their children had a median age of 6.0 months (IQR: 4.75, 8.25). The sample was racially and ethnically diverse, with 70% identified as White, 30% as Black, and 10% as Hispanic or Latinx. Most participants were married (65%) or living with a partner (90%), and just under one-third (30%) reported bedsharing with their child. The majority were breastfeeding (65%) and employed full-time (55%). Educational attainment was high, with 95% having completed education beyond high school (Table 1).

### Survey results

Survey responses indicated that participants consistently prioritized sleep-related strategies over those related to diet and physical activity. Among all the items rated, “improving sleep efficiency”—defined as getting more uninterrupted sleep—received the highest mean importance score (M = 3.90, SD = 1.07). Other highly rated sleep-related components included “reducing sleep-related worry” (M = 3.80, SD = 1.11), “establishing a sleep routine for yourself” (M = 3.70, SD = 0.86), and “helping your child sleep through the night” (M = 3.70, SD = 1.22). In contrast, items such as “reducing nightmares” (M = 2.05, SD = 1.27) and “reducing time in bed when not asleep” (M = 2.60, SD = 1.35) were rated lower in importance (Table 2).

While participants rated sleep-related strategies as highly important, survey results also revealed that several of these same items were among the most difficult to implement. “Reducing nightmares” (M = 3.45, SD = 1.05), “reducing time in bed when not asleep” (M = 3.35, SD = 1.09), and “improving sleep in complicated environments” (M = 3.25, SD = 1.25) were rated as particularly challenging. Even strategies that were rated as highly important, such as “helping your child sleep through the night” (M = 2.15, SD = 1.04) and “reducing sleep-related worry” (M = 2.65, SD = 0.81), were still considered more difficult than many diet- and activity-related strategies were.

### Interview Results

From our qualitative analysis, five themes emerged. (1) The participants affirmed that the newsletter reflected their experiences but identified gaps related to mental health and partner/provider support. (2) Sleep was viewed as the most important behavior to change, with strong support for strategies targeting efficiency, routines, and sleep-related worry. (3) Sleep is also perceived as the most difficult behavior to change due to infant-related sleep disruptions, parental fatigue, and limited knowledge of effective strategies. (4) Participants offered differing views on where to begin—some preferred starting with sleep, others with more feasible behaviors or an integrated approach. (5) Participants emphasized the need for support and accountability, including coaching, peer connection, and flexible tools.

#### Theme 1: The newsletter reflected participants’ experiences—but some aspects were missing.

Many participants reported that the newsletter accurately captured their postpartum experience. For example, one participant reflected, “*Um, pretty honest, um, it kind of summarizes, uh, the experience of a new mom pretty well, um, some of the feelings and experiences that happened in the first few months*. (ID: 49)” Relatedly, another participant said, “*So it says, uh, they need trustworthy sources of information…Hard…having time to exercise. A hard time getting enough sleep. Um, needing support to get sleep. Yeah. I think, like, all that is really important.”* (ID: 48)

However, the participants identified several areas they felt were missing or underemphasized. One participant reflected on the absence of honest discussion about the emotional and biological realities of the postpartum period, which impacts mental health:

“There’s not enough talk about how these healthy interventions like good sleep and eating well and exercise… how much they impact your already hormonal mindset. Your hormones are already playing such an ugly trick on you and making you feel sad and crazy and irrational and worried and anxious. But they don’t talk about how all of our needs are going unmet… and how that’s exacerbating all of those thoughts and feelings… I feel like if I would have the time to exercise and eat well and sleep, my mindset would be better.”

(ID: 49)

Another participant added, *“Helping people reduce their anxiety would be a good thing… not like diagnosed anxiety… there’s so much to worry about when you’re a new parent.”* (ID: 43) Another participant shared a similar perspective, “*Like, for me, I would say, like, just emotions and, like, mental–like, it talked about, like, worrying and concern… I don’t know if that’s necessarily, like, under the scope of all this to include*.” (ID: 48)

Others commented on systemic barriers to care and the lack of acknowledgment of broader contextual challenges, such as healthcare access and pandemic-related disruptions. *“There were longer waiting periods for providers for pregnant women… I didn’t feel like I was given modern solutions.”* (ID: 63)

#### Theme 2: Sleep was viewed as foundational to postpartum health and the most important behavior to change

Many participants described sleep as a foundational behavior—one that made it easier to engage in other health behaviors. As one participant explained, *“Getting, like, an uninterrupted night of sleep would be the most important over everything because, like I said, it’s like a—it’s like a domino effect.”* (ID: 49) Others emphasized that better sleep enabled them to be more present and productive: *“When I’m wellrested, I can just get more things accomplished in my day. I can cook. I have energy to clean. I have energy to be more active with her, more, like, present.”* (ID: 52)

Although some participants reported limited control over their bedtimes due to infant care, they expressed openness to the idea of creating personal wind-down routines. As one participant described,

“Establishing a sleep routine for myself. So when I have the opportunity for an uninterrupted sleep, like, being able to move into a routine that lets me and my body know it is time for bed, kick everything else, um– let everything move in and out of your head so that you can rest. Like, I think I need– I could, could use help with that.”

(ID: 51)

Another participant echoed this sentiment by stating, *“Learn some strategies to help establish a sleep routine for yourself. That’s a good one. Because if you don’t have proper rest, you’re fatigued, and you can’t care for your newborn… you need to be functional.”* (ID: 49)

#### Theme 3: Sleep was viewed as difficult to change due to infant demands and personal exhaustion.

The participants often pointed to infant care demands, unpredictable sleep patterns, and exhaustion as barriers to implementing sleep-related strategies. One participant noted,

“Probably—hmm, improving your sleep efficiency. That would be difficult. And that kinda ties in with helping your child sleep through the night… because she wakes up often, and that interrupts my sleep.” (ID: 62) Another shared, “All of the sleep ones really, like, both my child and me… would just be really hard.”

(ID: 39)

The participants also described how external factors often made sleep improvement feel out of reach. *“Helping your child sleep through the night… I think that’s next to impossible.”* (ID: 49) Others described the emotional effort required to improve sleep. *“When you’re trying to break a pattern, I think it takes a lot of energy, and it’s something that’s lacking at this time in my life.”* (ID: 62) One participant added, *“There’s nothing I can do sometimes to, like, make myself fall back to sleep.”* (ID: 39) These reflections suggest that many participants saw sleep as uniquely constrained by both external forces.

In addition to describing external barriers to improving sleep, such as infant unpredictability and fatigue, participants often expressed uncertainty about how to change their own sleep behaviors. For some, this lack of knowledge contributed to the perception that sleep was more difficult to improve than diet or activity was. As one participant admitted, *“I don’t—I don’t know anything that I can do to make it better… There’s nothing I can do to sleep. You know? There’s, there’s nothing I can do.”* (ID: 49) Others described relying on a limited set of strategies or not knowing where to start. *“I guess I don’t really know other things to try besides deep breathing.”* (ID: 45) One participant emphasized a desire to learn new approaches, stating, *“Everyone wants to think, like, yeah, that’d be really great if I could, you know, have ways or be taught interventions to, like, make these things happen.”* (ID: 56) These reflections suggest that improving sleep may require education about strategies that are realistic and actionable during the postpartum period.

#### Theme 4: Participants had differing views on where the intervention should begin

The participants were asked to reflect on which behavioral domain—sleep, diet, or physical activity—they felt would be the most appropriate starting point for a postpartum lifestyle intervention. The survey data revealed that sleep was consistently rated as the most important behavior to address, but the interviews revealed differing opinions on whether it should be the first focus of the intervention. As described above for theme 2, improving sleep could have a “domino effect” for improving other behaviors. Some participants reported starting with sleep because of its perceived impact on all other behaviors. One explained, *“I think the most important is, um, like, the sleep, ‘cause that’s gonna have a more positive effect on everything.”* (ID: 52) However, others felt that sleep was too difficult to tackle at the outset and preferred to begin with a behavior they found more achievable—such as dietary changes or increasing movement—as a way to build early success and confidence. One participant said, *“Once they [the child] start sleeping through the night and I can actually get more rest, then I can start making more changes[to their sleep].”* (ID: 61) Another added, *“It would be helpful to start with something I can actually do—then work toward the hard stuff like sleep.”* (ID: 54)

Some participants reflected on how different behaviors supported each other, suggesting a flexible or integrated approach rather than a fixed sequence. One participant shared, *“If I could just get a little more sleep, then I might have the energy to walk more or cook more meals. But I also know that moving around helps me sleep better, so maybe doing a little of both would help.”* (ID: 48) Others emphasized that all three behaviors—sleep, diet, and activity—were interconnected. As one participant explained, *“You can’t really do one without the other. Like, they work together.”* (ID: 50) Another participant described how physical activity supported multiple goals, noting, *“It helps with sleep. It helps with reducing stress… and even gives me a break from the kids.”* (ID: 21) Participants also expressed a desire to understand better how behaviors influence each other and to receive practical strategies that reflect this complexity.

#### Theme 5: Participants wanted more support and accountability to maintain healthy eating and exercise habits.

The participants emphasized the importance of support and accountability to maintain healthy lifestyle behaviors. Several described feeling more motivated when engaging with another person, such as a coach or peer. One participant explained,

“I know that I’m more motivated if somebody else is doing the activity with me… It’s harder for me to hold myself accountable for it. But, if I do it with someone or if I know that I’m going to be discussing it with someone else… then I will make it more so of a priority.”

(ID: 54)

Another expressed a need for guidance to help initiate new habits, sharing,

“Yeah. I would want, like, just someone to be like, ‘Okay. If you can do this for 30 minutes or this for 20 minutes or here’s some simple exercises just to, like, get you—’ ‘cause once I get on a, a routine, I’m normally pretty good. But it’s just getting on to that routine that’s challenging.”

(ID: 58)

Others highlighted the emotional value of connecting with people who shared similar experiences. As one mother shared, *“Some of [the stories] bring me to tears because it’s like I truly understand. It’s just nice to know that there is another woman out there that… brutally understands what you’re going through.”* (ID: 49)

In addition to interpersonal support, participants described technological tools as helpful for accountability. One participant suggested, *“So I think definitely notifications. Something where you could set notifications or, like, reminders to work out. Or maybe even notifications that give you, like, little tips about, you know, servings of fruits and vegetables.”* (ID: 50)

## Discussion

This study aimed to inform the development of Sleep Goal-focused Online Access to Lifestyle Support (GOALS), an integrated intervention addressing sleep, diet, and physical activity in the postpartum period, using the Diabetes Prevention Program (DPP) and the Transdiagnostic Sleep and Circadian (TranS-C) interventions as starting points. Guided by the program production stage of intervention mapping, we identified behavior change priorities, perceived barriers, and delivery preferences to refine the intervention content and structure. The participants affirmed the relevance of prior findings but emphasized important gaps—particularly the lack of attention to emotional and mental health, systemic barriers to care, and the need for trustworthy sources of information. They strongly endorsed sleep as foundational to postpartum health, citing its influence on mood, energy, and ability to engage in diet and physical activity. However, they also described sleep as the most difficult behavior to change due to infant-driven disruptions, maternal exhaustion, and uncertainty about effective strategies. The participants varied in their views on which behaviors the intervention should address first, with some preferring to start with sleep and others recommending a more flexible or staged approach. Across participants, there was a consistent call for support and accountability in interventions, delivered through both interpersonal connections and tools such as reminders or check-ins, and for interventions to offer flexible, self-paced engagement options that align with the realities of postpartum life.

This study’s results provide guidance on which components of the TranS-C intervention are most relevant for postpartum individuals. The participants consistently endorsed strategies such as improving sleep efficiency, establishing a sleep routine, and managing sleep-related worry as both important and, in some cases, feasible to implement. These findings confirm the relevance of the core components for postpartum individuals and offer valuable guidance for selecting optional modules most responsive to participants’ lived experiences. For example, optional modules focused on sleep efficiency and worry were highly endorsed, whereas modules such as reducing nightmares and addressing complicated sleep environments were less frequently identified as relevant. This underscores the value of tailoring the intervention to address the most salient sleep challenges for postpartum individuals while still anchoring the program in core behavioral strategies that support lasting change.

These findings align with existing qualitative research demonstrating the interconnectedness of sleep, diet, and physical activity among postpartum individuals. Previous studies have consistently shown that postpartum individuals view these behaviors as interrelated and influenced by common barriers such as fatigue, caregiving demands, and lack of time.(38–40) For example, Lim et al. identified psychological ability (e.g., mental exhaustion), limited social opportunity, and motivational trade-offs as major challenges to adopting healthy lifestyle behaviors in the postpartum period.(40) The participants reported that sleep deprivation limited their energy for physical activity and made planning or preparing healthy meals more difficult—themes that closely mirror our findings. Our study reinforces these patterns by showing that participants view sleep as a foundational behavior that shapes their ability to engage in diet and activity changes.

Our findings extend this work by identifying specific behavioral strategies that postpartum individuals prioritize and find actionable, such as improving sleep efficiency, establishing sleep routines, and reducing sleep-related worry. The participants in our study also highlighted the importance of integrated behavioral strategies, expressing interest in learning how behaviors interact. For example, participants wanted to understand how evening eating patterns and food choices affect sleep or how increasing physical activity might help them fall asleep more easily. While Lim et al. also emphasized the role of behavior regulation strategies (e.g., planning, prompts, goal setting), our results add to this by clarifying which specific sleep-related components participants want included and how they would like them delivered. Together, these findings support the development of flexible, low-burden interventions that reflect the lived experience of postpartum individuals and target behavioral interconnections to promote sustainable change.

A key strength of this study is the integration of both qualitative interviews and structured survey responses to identify not only what participants need but also what they believe is feasible. This mixed-methods approach provides a pragmatic framework for adapting evidence-based sleep content. However, the study also has limitations. The small and geographically limited sample size may affect the generalizability of the findings. While we included diverse voices, further research with larger, more representative samples is needed. Additionally, our reliance on self-reported perceptions of difficulty may not always align with actual behavior change outcomes. Importantly, because a behavior is perceived as difficult or less important, does not indicate that it should be excluded from intervention. Rather, these behaviors may require tailored messaging that connects them to participants’ goals or structured opportunities to try strategies they initially believe to be challenging. Our findings suggest that postpartum individuals are open to learning new approaches and may benefit from supportive, flexible opportunities to engage with content they might otherwise overlook. Future work should evaluate how perceived barriers and priorities translate into real-world intervention engagement and effectiveness and explore strategies for introducing difficult behavior changes in a motivating and empowering context.

These findings have several implications for intervention development. First, they support the inclusion of sleep strategies focused on sleep efficiency, routines, and worry management as core components of a postpartum lifestyle program. Second, the results suggest the value of flexible intervention design, allowing participants to begin with behaviors that feel most achievable. Third, the emphasis on the interconnection between behaviors reinforces the need for integrative content that addresses sleep, diet, and physical activity as mutually reinforcing domains. Given participants’ strong interest in support and accountability, interventions should also incorporate coaching, check-ins, or peer-based strategies to help sustain motivation. Finally, these insights can also be used to adapt the DPP for postpartum populations. While the DPP focuses primarily on caloric restriction and physical activity promotion for weight loss and maintenance,(41) our findings suggest that postpartum individuals are interested in understanding how diet and physical activity influence sleep, and vice versa. For example, participants expressed interest in learning how evening eating patterns or food choices affect sleep onset and quality, as well as how improving sleep could reduce late-night snacking or improve dietary choices. Similarly, the participants noted that physical activity helped them feel more alert during the day and improved their ability to fall asleep at night. These reciprocal relationships provide an opportunity to enhance the DPP’s content by explicitly incorporating strategies that leverage the interdependence between sleep, diet, and physical activity to promote sustainable behavior change.

## Conclusion

In conclusion, this study provides a participant-informed foundation for developing an integrated postpartum intervention that addresses sleep, diet, and physical activity. First, sleep emerged as the highest-priority behavior for participants but also one of the most difficult to change, underscoring the need for supportive, flexible strategies. Second, participants identified specific sleep-related components —such as improving sleep efficiency, reducing sleep-related worry, and establishing routines—as both relevant and feasible, guiding the selection of core and optional intervention modules. Third, while participants viewed sleep as foundational, many valued the opportunity to begin with diet or activity behaviors that felt more attainable, highlighting the importance of offering tailored sequences. Fourth, the participants emphasized a strong desire for accountability and connection, suggesting that interventions should include coaching, check-ins, or peer support. By centering on postpartum individuals’ experiences and priorities, this study offers practical guidance for designing acceptable and effective interventions that address the real-world challenges of postpartum recovery and behavior change.

## Supplementary Material

Supplementary Files

This is a list of supplementary files associated with this preprint. Click to download.

- Appendix1NewsletterUpdate.pdf
- Appendix2importancedifficulty.docx
- Appendix3interviewguide.docx
- Appendix4COREQChecklistCompleted.docx

## Figures and Tables

**Table 1:** Sample descriptive characteristics

Characteristic	N = 20^1^
Age	33 (29, 35)
Child’s age	6.00 (4.75, 8.25)
Kids in the home	
1+	12 (60%)
None	8 (40%)
Married, yes	13 (65%)
Live with a partner, yes	18 (90%)
Bedsharing with partner, yes	18 (90%)
Bedsharing with child, yes	6 (30%)
Breastfeeding infant, yes	13 (65%)
Formula feeding infant, yes	11 (55%)
Feeding infant other foods, yes	2 (10%)
Education Attainment	
High school	1 (5.0%)
Greater than high school	19 (95%)
Working status	
Stay at home	6 (30%)
Part-time	3 (15%)
Full-time	11 (55%)
Self-reported race and ethnicity	
White, yes	14 (70%)
Black, yes	6 (30%)
Hispanic/Latinx, yes	2 (10%)
All other racial identities, yes	1 (5.0%)

1 Median (IQR); n (%)

**Table 2:** Participant ratings of difficulty and importance of potential intervention components

Survey item	Mean (SD)
Difficulty	
Reduce nightmares	3.45 (1.05)
Reduce time in bed	3.35 (1.09)
Adjust bedtime	3.25 (1.07)
Sleep environments	3.25 (1.25)
Self-sleep routine	2.70 (1.08)
Sleep for diet	2.70 (0.92)
Diet for sleep	2.65 (0.99)
Reduce sleep worry	2.65 (0.81)
Activity for sleep	2.45 (0.94)
Sleep efficiency	2.40 (1.10)
Sleep weight	2.30 (0.92)
Child sleep	2.15 (1.04)
Sleep for activity	2.15 (0.81)
Importance	
Sleep efficiency	3.90 (1.07)
Activity for sleep	3.80 (0.77)
Diet for sleep	3.80 (0.77)
Reduce sleep worry	3.80 (1.11)
Child sleep	3.70 (1.22)
Self sleep routine	3.70 (0.86)
Sleep for activity	3.60 (0.82)
Sleep weight	3.60 (0.94)
Sleep for diet	3.45 (0.89)
Adjust bedtime	3.20 (1.01)
Sleep environments	2.95 (1.39)
Reduce time in bed	2.60 (1.35)
Reduce nightmares	2.05 (1.27)

Mean values represent average participant ratings on a scale from 1 (Not at all) to 5 (Extremely).

## Data Availability

De-identified interviews can be made available with a data use agreement.
